# Tabular strategies for metadata in ecology, evolution, and the environmental sciences

**DOI:** 10.1002/ece3.9245

**Published:** 2022-08-25

**Authors:** C. J. Lortie, Camila Vargas Poulsen, Julien Brun, Li Kui

**Affiliations:** ^1^ National Center for Ecological Analysis and Synthesis, UCSB Santa Barbara California USA; ^2^ Department of Biology York University Toronto Ontario Canada; ^3^ Marine Science Institute, UCSB Santa Barbara California USA

**Keywords:** data, metadata, open science, R programming language, tables, template, workflows

## Abstract

Data support knowledge development and theory advances in ecology and evolution. We are increasingly reusing data within our teams and projects and through the global, openly archived datasets of others. Metadata can be challenging to write and interpret, but it is always crucial for reuse. The value metadata cannot be overstated—even as a relatively independent research object because it describes the work that has been done in a structured format. We advance a new perspective and classify methods for metadata curation and development with tables. Tables with templates can be effectively used to capture all components of an experiment or project in a single, easy‐to‐read file familiar to most scientists. If coupled with the R programming language, metadata from tables can then be rapidly and reproducibly converted to publication formats including extensible markup language files suitable for data repositories. Tables can also be used to summarize existing metadata and store metadata across many datasets. A case study is provided and the added benefits of tables for metadata, a priori, are developed to ensure a more streamlined publishing process for many data repositories used in ecology, evolution, and the environmental sciences. In ecology and evolution, researchers are often highly tabular thinkers from experimental data collection in the lab and/or field, and representations of metadata as a table will provide novel research and reuse insights.

## Graphical abstract

The last few years have pushed many disciplines to additional computational work. In ecology and evolution, we have embraced and used computation extensively including R, Python, open data, and many other open science tools. Data reuse even within existing collaborative networks can be a challenge nonetheless, and better metadata is the one outstanding issue. Here, we highlight this continued opportunity to improve as a discipline anew. We also provide a novel solution—consider using tables for metadata.

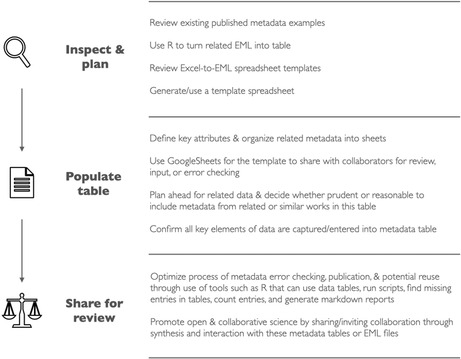

## 1 INTRODUCTION

Replication and reuse are increasingly common, legitimate, and critical forms of science in ecology, evolution, and the environmental sciences. Reuse of evidence is a function of scientific syntheses including those that leverage data (Halpern et al., 2020). There are at least three forms of ecological synthesis science including those that compile data, methods, and/or theory (Halpern et al., 2020). In both primary and synthesis science, this is often described as reproducible science because the goal of our work including code and data can be reused and replication of an experiment or theory in a novel/alternative context (Alston & Rick, 2021; Shaw et al., 2016). Data are a currency and cornerstone of ecology and evolution. In the environmental sciences, data are also commonly used to enable reproducible science (directly via data reuse and indirectly through conceptual replication) to explore challenges (Giuliani et al., 2019; Li, 2019). Computational biology in all these fields similarly assumes that research is accelerated and supported by standardized and precise metadata (Leipzig et al., 2021). Metadata are the descriptions and information that describe the data (Jones et al., 2001; Reichman et al., 2011). If one elects to publish the data once the experimental work is complete (but perhaps before a paper is published if this is one of the goals of a project), this can also enable a novel workflow because in writing the metadata for a dataset one is better prepared to write formal methods, identify gaps, and more deeply consider differences between the final data collected from what was initially planned (Lortie, 2021). Reading the metadata from another project even more clearly illuminates the dire need for better metadata (Edwards et al., 2011), and additional attention to metadata is thus merited. More transparent and networked science using this framework is a highly likely outcome.

We propose that the “data” component of the metadata be more directly examined in contemporary ecology and evolution by treating metadata as tables. Metadata are a form of scientific evidence and thus a valid open scientific object as well (Boettiger, 2019). The value of metadata, attention to its use, and its relative transparency to the associated data have been examined conceptually in other fields including digital forensics (Alanazi & Jones, 2015), medicine (Sakai, 2001), and social studies (Mayernik, 2019) to name a few disciplines. We have examined metadata in depth in ecology and evolution as well (Michener, 2015) but less frequently from a process‐based perspective as developed in other fields (but see, for instance, Leinfelder et al., 2011; Mena‐Garcés et al., 2011). Metadata as a process includes moving metadata from forms and fields (in some capacity) to make it more visible (Alanazi & Jones, 2015), using metadata as a mechanism to iteratively and positively evaluate accountability in the data (Mayernik, 2019), and as a form of evidence that encodes the schema or approach of a study for novel perspectives to other practitioners (Sakai, 2001). These processes and many other similar uses for metadata of course do commonly occur in our domain including using metadata as a framework to design field data ingestion and collection (Jones et al., 2007). We are not proposing that ecologists ignore structured metadata such as ecological metadata language, i.e., EML (Fegraus et al., 2005)—on the contrary, these standards are necessary to fully capture the complexity of describing data and thus increase value in data. Nonetheless, there is still room to innovate on the ease of both the creation and reuse of metadata for scientists through very simple and intuitive changes in practice and how we structure and inspect metadata to better learn and do science. Given the relatively high frequency of use of scripting languages such as R to handle data in ecology and evolution (Lai et al., 2019), we can further explore metadata through computational workflows in many instances particularly when open science methods are used.

Tables are tools. Information organized into tables such as after the metadata are published or before as a mechanism to document it and prepare for publication is formative for at least several principal reasons. Tables can function as cognitive tools because they provide concrete, logical representation of information including numbers, text, annotations, and other objects even images (Marti, 2009; Reuter et al., 2022). Tables can also be used as mental models that blend freeform information with more rigid or fixed information, i.e., like metadata (Mangano et al., 2011). Hence, we advance that “tabular thinking” can thereby enable cognitively mapping and organizing ideas, increase comprehension and retention, and can function as a model to aggregate mixed evidence including annotation into one place. Additionally, tables can facilitate decision‐making by providing information in parallel representations versus serial—ideas all lined up so to speak (Cappella et al., 2016). Even “untidy” tables with comments, annotations, and work‐in‐progress markup have been proposed as highly effective tools because spreadsheets are a fundamental component of the information ecosystem in working with data including inspecting and interacting with the evidence (Bartram et al., 2021). It is useful to inspect data in more than one form from tables to plots.

Ecologists, evolutionary biologists, and many environmental scientists interact with the data in a table, perhaps in a spreadsheet, at some point in their workflows. Treating metadata as data in tables will thus enable a more tangible and parallel or sympatric view of the attributes of both the data and metadata. It can also enable cross contrasts between datasets and opens up research, reuse, replication, and work with metadata if provided in a table (Bilalli et al., 2016; Willis et al., 2012). These processes can happen without tables but using them can increase the ease of these endeavors for many through the benefits of logic, clarity, and organization of information into the rows, columns, and sheets of tables. The R programming language (R‐Development‐Core‐Team, 2022) and particularly the set of packages within this environment entitled the “tidyverse” strongly leverages data in tables and their strengths within this computational environment (Wickham et al., 2019). In R, dataset up as data tables are called dataframes (or tibbles in the tidyverse). This enables facile manipulation, handling, extraction, and inspection including simple summaries of the dataframe. The tidy data philosophy promotes a structured approach and the formatting of evidence necessarily includes decisions on whether evidence should be formatted as wide or long and how we then map ideas onto rows as independent observations of a process (Wickham, 2014). This logic and clarity align with functional programming and thinking because tables coerce one into decisions about the specific meaning of an attribute and where it should be placed with the table relative to other information. It also applies to metadata because we can observe patterns in the metadata particularly when we have more than one dataset to publish. This framework and its benefit also strengthen replicability because it can be used to generalize metadata for projects and sets of experiments. We can thus use R and other tools to streamline publishing and working with the metadata. An example will consolidate both the conceptual and practical implications of metadata in tables.

## 2 EXAMPLE

There are many examples of metadata as data tables. A brief list includes several cogent examples at different points in a data science workflow for ecology and evolution (Table 1). The current relevant package offerings in R fundamentally either work with existing metadata, then tabularize, or conversely, convert tables into ecological metadata language, i.e., EML (Fegraus et al., 2005; Jones et al., 2001). EML defines a controlled, high‐quality machine‐readable format that organizes the rich and varied metadata content common in ecology and evolution (Gil et al., 2011). Tables can also be used to store existing published metadata, post hoc, for scientific syntheses (Willis et al., 2012). These three potential workflows describe a logical framework for tabular strategies in process‐based use of EML and metadata in our fields (EML to tables, tables to EML, or tables to organize and store existing metadata). Here, we highlight a prescient example for The Environmental Data Initiative (data) repository, i.e., EDI (Gries et al., 2019), because it supports the framework proposed that tables for metadata will streamline processes and promote a novel workflow. This example uses a template in spreadsheet format first and thereby consolidates and makes tangible many instructive components of a dataset including gaps when metadata are absent or not reported. There are already two main approaches to submitting data currently listed on the instructions for EDI (https://environmentaldatainitiative.org). A form‐based online tool entitled “ezEML” or an EDI template as an MS Word file for metadata that one completes and submits with data to the data preservation team. Both adhere to EML and encourage best practices in annotating data through metadata.

**TABLE 1:** A list of representative R packages associated with ecological metadata language (EML) standards and tabular thinking framework.

Tool	Description	Primary use	Functions	Tabular strategy	Source
EMLassemblyline	R package to create EML metadata for dataset publication	Generate metadata	Generate EML using content from a single‐use input or shiny interface	Tables to EML: input information provided in Word document tables into an R script to create a formatted EML (XML extension)	https://github.com/EDIorg/EMLassemblyline
Excel‐to‐EML	R package to enter metadata from an MS Excel template	Generate metadata	Three R functions support reading information from Excel into metadata for EDI data repository publication	Tables to EML: Excel tables store and organize metadata for subsequent EML creation	https://github.com/lkuiucsb/Excel‐to‐EML
LTER‐core‐metabase	PostgreSQL‐based relational database model designed for the management of ecological metadata	Generate metadata	Metadata database used to store attributes	Tables to organize metadata: database used to store and organize metadata in tabular format	https://github.com/lter/LTER‐core‐metabase
MetaEgress	R package to create Ecological Metadata Language (EML) standard metadata documents from an installed and populated LTER‐core‐metabase	Generate metadata	The main functions are to query metadata LTER‐core‐metabase, then insert information into appropriate EML slots, then output an R list structured according to the EML standard	Tables to EML: read metadata from the LTER‐core‐metabase output tables and generate EML file	https://github.com/BLE‐LTER/MetaEgress
metajam	Resources to download specific datasets and their associated metadata from DataOne	Reuse metadata	Download data using R including the associated metadata	EML to tables: serialize EML into R dataframes	https://cran.r‐project.org/web/packages/metajam/index.html
pkEML	R package to convert Ecological Metadata Language documents to tables to help data managers migrate their metadata archives. Can also be used for meta‐analaysis	Manage and reuse metadata	Functions tabularize existing EML and support project work	EML to tables: extract contents from corresponding xml nodes and populate R dataframes	https://github.com/atn38/pkEML

*Note*: Offerings available on both CRAN and GitHub are listed. The tool is the formal package name, and the description is a short statement of the goal for the resource. The primary use column lists the main purpose of the resource. The functions column describes some of the utilities of each resource in working with metadata and tables at some point on a workflow of publishing or interacting with published datasets in ecology and evolution. The tabular strategy underscores the three strategies evident to date including tables of metadata to EML, EML to tables, and finally tables to organize existing metadata. The source column provides the most current location for installation of each specific resource.

Nonetheless, a third table option using an MS Excel template and R has been developed entitled “Excel‐to‐EML” (Kui, 2022) that features all of the benefits of considering tabular strategies for metadata. These R‐structured resources provide a working directory workflow with an Excel template, two examples, and three R functions (GitHub repository: https://github.com/lkuiucsb/). The Excel template is used for collecting and storing metadata for the dataset and project‐related attributes. These details include the following information: dataset title, personnel, keywords, data column description, temporal and spatial coverage, and project funding information. The three R functions were designed to automate the EML generation process via three intuitive steps. First step and function, read the metadata content from the Excel template with the “get_meta_xlsx” function. This function extracts the cell contents from the Excel template and merges them into a list of data tables indexed by the dataset ID (numerical values that the user provides). Second step, construct the EML document for the designated data package (for each dataset ID) using the “generate_EML_Assemblyline” function. This function filters the data tables to keep the dataset‐specified content, and it then assembles an EML document adhering to both the current EML 2.2.0 specifications and also the proposed best practices for ecological metadata (Jones et al., 2019). The abstract and methods for the data package are also read from MS Word documents provided by the user and converted into text type to insert as EML nodes (Boettiger et al., 2022). Third and final step, a function exports the EML document using R entitled “write_eml_excel.” This function writes out/generates a .xml file and runs a list of EML validation checks. In the case of invalid EML such as missing metadata information, warning or error message(s) will appear in the console window. Two examples were presented in the package within distinct project folders (Kui, 2022). The first example provided in this package is a series of plant architecture parameters (i.e., plant height, diameter, etc.) that were measured on cottonwood and tamarisk seedlings. The second example is a kelp frond count in the Santa Barbara Channel. These sample data packages accommodate the most common formats of data tables (in csv format). Both include data entities such as R scripts or PDF documents that are frequently packaged together with the research data in ecology and evolution published data packages. When describing the dataset attributes, the data packages also present four column/vector classes in R dataframes including character, numeric, date, and categorical (the latter requires an additional definition for each of the factors). The data package from the plant architecture parameters was published in EDI (Kui et al., 2018). This is a comprehensive and well‐developed set of resources to explore. It is accessible to ecology and evolution scientists because it represents most of the challenges that we tackle in organizing primary field or lab research into metadata.

The workflow can also be innovated further to include reporting in R Markdown documents (https://rmarkdown.rstudio.com) for attribute summary gaps that need to be completed before publishing the data. This is an additional strength of treating metadata as tables and dataframes and working in an R environment—RStudio functionality can be leveraged. The Palmyra Atoll Data Library (PADL, https://github.com/padl‐project) is an adaptation of the Excel‐to‐EML workflow (Kui, 2022) using R Markdown to facilitate the process of documenting data that has historically lived only in the computers of individual researchers. The goal of PADL is to document and publish data collected at Palmyra Atoll over the last two decades and into the future. Using the MS Excel metadata template, PADL gathers all necessary metadata and efficiently processes the metadata into EML format to publish data packages into the Environmental Data Initiative repository by a data manager and software engineer. Two very different examples of high‐quality metadata (with data) from the PADL initiative have been published at EDI (Guerra et al., 2022; Wegmann & Alifano, 2022) using a workflow adopted by the data manager. Metadata from each published package to date was compiled into a singular, global tabular form. This enables project‐level management and record tracking of the published information. Using tables to collect metadata also makes it easier for the individual data owners to document the metadata and error check. The data manager is also able to oversee the collective metadata under a larger project through tabular strategies of compiling and sharing the metadata. Tables can provide the big picture for a project with distributed datasets.

If the number of datasets increases significantly within a research group or regional research institution, a highly structured PostgresSQL relational database (i.e., an open‐source object‐relational system) can also be used for metadata collection and storage such as the one used by The National Science Foundation Long Term Ecological Research Network (https://github.com/lter/LTER‐core‐metabase). The database can be used in tabular form for convenience, but more importantly, it has multi‐level controls for preventing errors or duplicates and can serve as a backend for a website. Tables for metadata can connect researchers in networks. Tables can also function as templates to enable clear thinking, reporting, and better documenting of metadata. Tables thus connect evidence, ideas, and people in our field—potentially as stand‐alone or linked research objects (Boettiger, 2019)—particularly if the culture of more open science continues to develop and evolve.

## 3 IMPLICATIONS WITH BEST PRACTICES ENABLED FROM TABULAR STRATEGIES FOR METADATA

Metadata are like the methods for an experiment in a brief, annotated structure. Metadata describe datasets or deployed real‐time measuring processes in an ecosystem such as sensor arrays. The units, scale, duration, location, and many other salient experimental design decisions are collective components that all standard metadata languages capture. We advance open and replicable science through complete and comprehensive metadata. Sharing data through data repositories that use common metadata standards for one's field is thus a prudent strategy. Metadata without the data can also be published. A simple step at some point in the workflow of data curation or generating metadata, if not present already in existing practices, is the development of a table for the metadata. Tables provide tools for cognitive analyses, computational work in environments such as R, and the means to develop templates for teams. Tables are a tangible representation of metadata in a format more accessible than markup files, lists, forms, or distributed entry fields. Tables provide the further benefits of mental models and a concrete, logical representation of all the information that can comprise metadata in one place. To capitalize on these benefits, metadata in tables is recommended, and a major implication is that this framework informs new scientific workflows.

**FIGURE 1:**
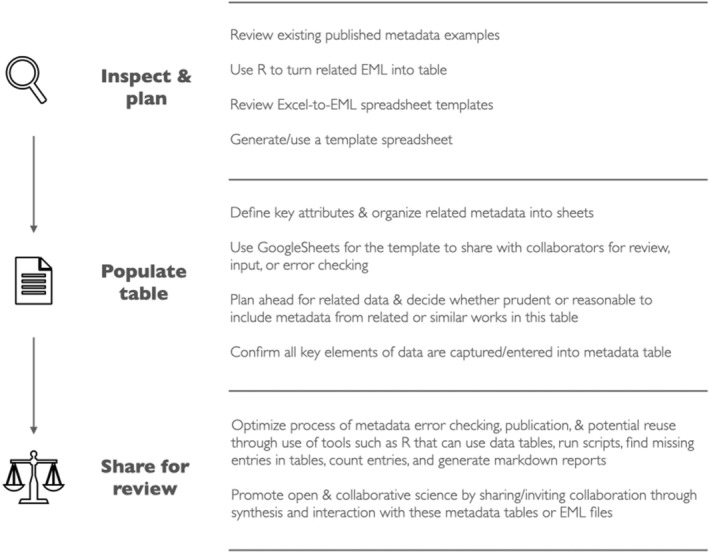
A general workflow for using tables to support better metadata in ecology and evolution. Three high‐level steps are proposed as a simple heuristic. Each step includes details to consider for the general process of developing well‐articulated structured metadata for publication in a data repository. The open‐source programming language R is listed in the details alongside ecological metadata as examples. However, other tools and standards can be sourced at each step depending on the data and methods. The final step share and review will support iteration of the process for others if the metadata tables are published openly.

A very high‐level, abstract workflow that summarizes the principles and benefits of metadata in one place for existing R packages that source or generate tables (including the Excel‐to‐EML example) is provided here (Figure 1). The workflow describes how to implement a general tabular strategy for metadata, and it is intended as a simple visual heuristic. This resource is a descriptive snapshot of tabular strategies for metadata and not prescriptive—innovate and use components as needed to treat metadata as a process and collaborative research opportunity. These three overarching steps here extend and generalize the Excel‐to‐EML workflow if you are considering another data repository in addition to EDI. The first step, inspect and plan, proposes that a cursory review of existing published metadata will improve your metadata. If metadata are published as EML and not tables, use one of the R tools summarized here to convert to tables (Table 1). Inspecting relevant metadata particularly in tables from related studies provides a clearer vision of your specific metadata. The scientific communication analog would be that reading related publications and checking journal requirements, styles, and contribution formats for papers prior to initiation of writing up a study for publication is common practice. The same principle applies to metadata. Even a little a prior reading and review will advance more efficient metadata writing that is better structured for a template table and for subsequent publication.

The second step, populate table, captures most of the steps provided in the Excel‐to‐EML package including enter metadata into a table, get the information from tables into EML and error check, and prepare/export the tabular data into the metadata languages as an xml file for publication. We extend this by suggesting that one must consider how to structure a template or table when developing from scratch (sheets vs. additional columns for instance) and that online spreadsheets can accelerate input and review by contributors. This is also a good point in your workflow to ensure that the salient elements of design adhere to EML best practices and reflect standards and norms in the field from similar published instances.

These latter steps can also be extended into the third high‐level step proposed herein, share for review, with collaborators examining warnings, missing entries, and errors, ensuring that all information is representative of the project. This step can also include optimization if one has several related datasets to publish. Scripts that are developed that summarize, report missing entries, or generate reports including visualizations can be explored and published alongside the data and metadata in all data repositories. This final step supports synthesis and community‐wide scientific knowledge development because metadata tables and well‐articulated EML files shared publicly can feed back into the very first step again for new researcher reuse. This process‐based framework reflects learning in ecology and evolution including how we use data science, computation, and scientific communication to support one another.

The practical implications of this workflow are diverse. Tables are easier for customization and modification than many metadata file formats. Research group‐specific attributes can be added to the table such as multiple missing values for the column. Tables accommodate EML content but can be extended to host ISO standard xml or other metadata languages (Smith & Schirling, 2006). Tables are flexible for partial metadata collection for simple projects. It is easy to organize repeated information (cut and paste). Practically, understanding how to complete each cell in a table can still be challenging at the onset even if all contributors participated in data collection. The first step of plan and review reduces this friction. Additionally, one can develop a handbook/manual for the metadata table for your network. Filling in all metadata for each dataset can be missed at times because contributors miss the tabs/sheets in the template (similar to what happens with data in team tables). Remind the contributors to review all sheets. Metadata in any well‐structured common format is critical, but streamlined work is a valuable aspiration. However, with a little effort, we can go a long way to better comprehension and retrievability with refinements through metadata in tables. Pragmatically, tables expand the contributor base in our fields to open metadata through accessibility/familiarity with the format and support current researchers who write EML with scope to optimize, share, and develop new tools.

## AUTHOR CONTRIBUTIONS

**Camila Vargas Poulsen:** Conceptualization (equal); data curation (equal). **Julien Brun:** Conceptualization (equal). **Li Kui:** Conceptualization (equal); data curation (equal).

## CONFLICT OF INTEREST

The authors declare no conflict of interest financial or otherwise.

### OPEN RESEARCH BADGES

This article has earned Open Data and Open Materials badges. Data and materials are available at https://doi.org/10.6084/m9.figshare.14773506.v1.

## Data Availability

All data are published and openly accessible (Guerra et al., 2022; Kui et al., 2018; Wegmann & Alifano, 2022).
